# P3MA: A Promising Mycobacteriophage Infecting *Mycobacterium abscessus*

**DOI:** 10.3390/antibiotics14080801

**Published:** 2025-08-06

**Authors:** Antonio Broncano-Lavado, John Jairo Aguilera-Correa, Françoise Roquet-Banères, Laurent Kremer, Aránzazu Mediero, Mateo Seoane-Blanco, Mark J. van Raaij, Israel Pagán, Jaime Esteban, Meritxell García-Quintanilla

**Affiliations:** 1 Clinical Microbiology Department, IIS-Fundación Jiménez Díaz, Autonomous University of Madrid, 28040 Madrid, Spain; antonio.broncano@quironsalud.es (A.B.-L.); jesteban@fjd.es (J.E.); 2 Institut de Recherche en Infectiologie de Montpellier, Centre National de la Recherche Scientifique UMR 9004, Université de Montpellier, 34293 Montpellier, France; john.aguilera@fjd.es (J.J.A.-C.); francoise.roquet-baneres@irim.cnrs.fr (F.R.-B.); laurent.kremer@irim.cnrs.fr (L.K.); 3 CIBERINFEC-Consorcio Centro de Investigación Biomédica en Red (CIBER) de Enfermedades Infecciosas, 28029 Madrid, Spain; 4 Institut National de la Santé et de la Recherche Médicale (INSERM), Institut de Recherche en Infectiologie de Montpellier, 34293 Montpellier, France; 5 Bone and Joint Research Unit, IIS-Fundación Jiménez Díaz, Autonomous University of Madrid, 28040 Madrid, Spain; aranzazu.mediero@iis-fjd.es; 6 Department of Macromolecular Structure, Centro Nacional de Biotecnologia, Consejo Superior de Investigaciones Científicas, 28049 Madrid, Spain; mateosb1992@gmail.com (M.S.-B.); mjvanraaij@cnb.csic.es (M.J.v.R.); 7 Centro de Biotecnología y Genómica de Plantas UPM-INIA/CSIC and E.T.S. Ingeniería Agronómica, Alimentaria y de Biosistemas, Universidad Politécnica de Madrid, 28223 Madrid, Spain; jesusisrael.pagan@upm.es

**Keywords:** phage therapy, mycobacteriophage, *Mycobacterium abscessus*, biofilm, granuloma, antibiotic

## Abstract

Background/Objectives: *Mycobacterium abscessus* is an opportunistic pathogen causing infections mainly in patients with immunosuppression and chronic pulmonary pathologies. Extended treatment periods are needed to tackle this pathogen, bacterial eradication is rare, and recurrence can take place with time. New alternative treatments are being investigated, such as bacteriophage therapy. This work describes the characterization of the mycobacteriophage P3MA, showing its ability to infect clinical and standard *M. abscessus* strains. Methods: Phylogenetic analysis, electron microscopy, growth curves, biofilm assays, checkerboard, and granuloma-like medium studies were performed. Results: P3MA inhibited the growth of clinical samples in both planktonic and biofilm states as well as in a granuloma-like model. The study of the interaction with antibiotics revealed that P3MA exhibited an antagonistic effect combined with clarithromycin, indifference with amikacin, and synergy with imipenem. Conclusions: All these results suggest that, after genetic engineering, P3MA could be a promising candidate for phage therapy in combination with imipenem, including lung infections.

## 1. Introduction

*Mycobacterium abscessus* (*M. abscessus*) is an environmental rapidly growing mycobacterium [1]. The species is currently divided into three subspecies: *M. abscessus* subsp. *abscessus*, *M. abscessus* subsp. *bolletii*, and *M. abscessus* subsp. *massiliense* [2,3]. This bacterium is an opportunistic pathogen that causes infections primarily in the lungs [4].

The main risk factors for *M. abscessus* infections are immunosuppression and chronic pulmonary pathologies, such as bronchiectasis and cystic fibrosis, among others. It is commonly associated with other bacteria, such as *Pseudomonas aeruginosa* [4]. Once *M. abscessus* reaches inside the human body, it can be phagocytosed by host macrophages. However, the bacteria can resist intracellular destruction and induce the production of proinflammatory cytokines and tumor necrosis factor [5] so that the host immune system will recruit new B and T lymphocytes to the site of infection [6]. At this point, a dynamic structure develops between the host and the mycobacterium, known as a granuloma, which is a hallmark of mycobacterial infections [7].

*M. abscessus* has two different colony morphotypes, smooth and rough, depending on the presence or absence of glycopeptidolipids (GPL), respectively. Smooth colonies have a convex, opaque, and uniform appearance, while rough colonies are flat and cordate [8]. The transition appears to proceed from smooth to rough morphotypes during infection, and the rough phenotype is associated with more severe and persistent infections [9,10]. Like other bacteria, *M. abscessus* survives different environmental adversities, such as lack of nutrients, as part of bacterial communities encapsulated in an extracellular matrix secreted by the bacteria, known as biofilms [11], which are different from granulomas.

In addition to granuloma formation and biofilm production, *M. abscessus* is intrinsically resistant to many antibiotics [12]. Most recommendations are directed at the treatment of pulmonary disease, which consists of an induction phase with three or four antimicrobials (one or two of them intravenously administered) and a suppression phase with two oral or inhaled antimicrobials, depending on the antibiotic susceptibility of the strain and tolerance of the antibiotics over extended treatment periods [13,14].

Numerous investigations have been carried out in recent years to establish new alternative therapies to treat *M. abscessus* infections, including the use of nanoparticles, antimicrobial peptides, phototherapy, antibiofilm agents, and bacteriophage therapy [15]. Mycobacteriophages are viruses that use mycobacteria as hosts. There are more than 14,000 mycobacteriophages, and most of them were isolated against *Mycobacterium smegmatis*. Over 2500 mycobacteriophages have been sequenced and analyzed [16]. According to their sequence homology, they are grouped into clusters, being further divided into subclusters and singletons, and over one-half of the clusters contain an integration cassette [17]. On the other hand, the *M. abscessus* prophages are assorted into clusters named MabA, MabB, etc. To date, all characterized mycobacteriophages only infect and efficiently kill rough strains [18].

Cases of personalized treatment using mycobacteriophages are scarce [15,19,20,21], and many of them include genetic engineering to exclude toxins and lysogeny. Therapy using lytic phages is promising in patients with prolonged or recurrent infections. There is a lack of mycobacteriophage characterization in the literature, especially their effect in biofilms, granulomas, or in vivo. To date, only one study describes the effect of *M. abscessus* phages against biofilms and includes combinations with antibiotics [22], and only one work analyzes the effect of two phages in an animal model (zebrafish) [23]. Moreover, there is a study gap in phage–antibiotic interactions. This work aimed to characterize the mycobacteriophage P3MA in vitro, testing its capacity to infect *M. abscessus* clinical strains, to evaluate the effect alone and combined with antibiotics using the checkerboard method, which tests a wide range of combinations, and the efficacy against rough strains in both planktonic and biofilm states as well as in a granuloma-like model.

## 2. Results

### 2.1. P3MA Is a Siphovirus with 17 Sequences Genetically Near and Stable at Physiological Conditions

**Figure 1:**
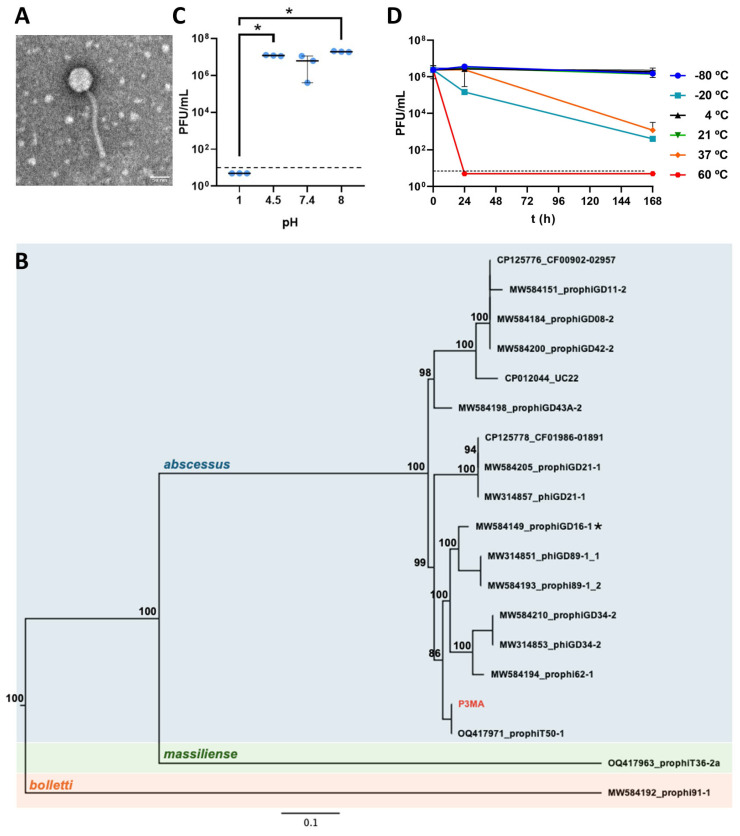
Characterization of P3MA. (**A**): Morphology of the P3MA mycobacteriophage. (**B**): Maximum likelihood phylogenetic tree constructed using full-length genomic sequences of *M. abscessus* phages. Node numbers indicate bootstrap values in percentages (1000 pseudo-replicates). P3MA, isolated and characterized here, is indicated in red. Phage sequences obtained from *M. abscessus* subsp. *abscessus* (blue), *M. massiliense* (green), and *M. bolletii* (orange) are shaded. An asterisk indicates the phage extracted from *M. massiliense* clustering with *M. abscessus* phages. The tree is midpoint rooted. (**C**): pH stability of P3MA for 1 h at pH 1, 4.5, 7.4, and 8 in SM buffer. Control was bacteria in the culture medium. The bars represent the median and the interquartile range. (**D**): P3MA temperature stability. Incubation for 24 h and 168 h at temperatures of −80 °C, −20 °C, 4 °C, 21 °C, 37 °C, and 60 °C. The bars represent the median and the interquartile range. The horizontal dashed lines represent the limit of detection (10 PFU/mL).

To visually identify the mycobacteriophage, P3MA was imaged in an electron microscope (Figure 1A). The phage P3MA showed a 65 ± 3 nm (n = 10) wide capsid and a 179 ± 7 nm (n = 10) long tail. The tail was long, flexible, and narrow, indicating that the phage is a siphovirus of the Caudoviricetes class.

Seventeen nucleotide sequences were identified as the genetically nearest to P3MA (Figure 1B). The majority of these sequences were isolated from *M. abscessus* subsp. *abscessus*, but two phage sequences were obtained from *M. abscessus* subsp. *massiliense*. P3MA clustered with one sequence belonging to the prophage T50-1 (which is part of the Cluster MabB), sharing 99% of nucleotide identity, which was retrieved from *M. abscessus* strain T50, as we previously described [24]. This strain is the most diverged subsp. *abscessus* strain from the reference strain ATCC19977 [25]. Accordingly, P3MA shared between 85 and 96% nucleotide identity with phages isolated from other strains of this subspecies (Figure 1B). Phages from subsp. *massiliense* and *bolletii* were phylogenetically more distant, except for phage GD16-1 from subsp. *massiliense*, which clustered with phages from subsp. *abscessus* (Figure 1B).

The isolated phage was stable at pH 4.5, 7.4, and 8 after a 1 h exposure; however, at pH 1, no bacteriophage was present in 100 µL (Figure 1C). Regarding thermal stability, P3MA was stable at most of the temperatures tested (21 °C, 4 °C, and −80 °C) after 24 h or 168 h of exposure, although at –20 °C and 37 °C, significant differences were observed. In turn, P3MA was unstable at 60 °C from the first point tested (24 h) (Figure 1D).

### 2.2. P3MA Infects Different Clinical M. abscessus Strains

**Figure 2:**
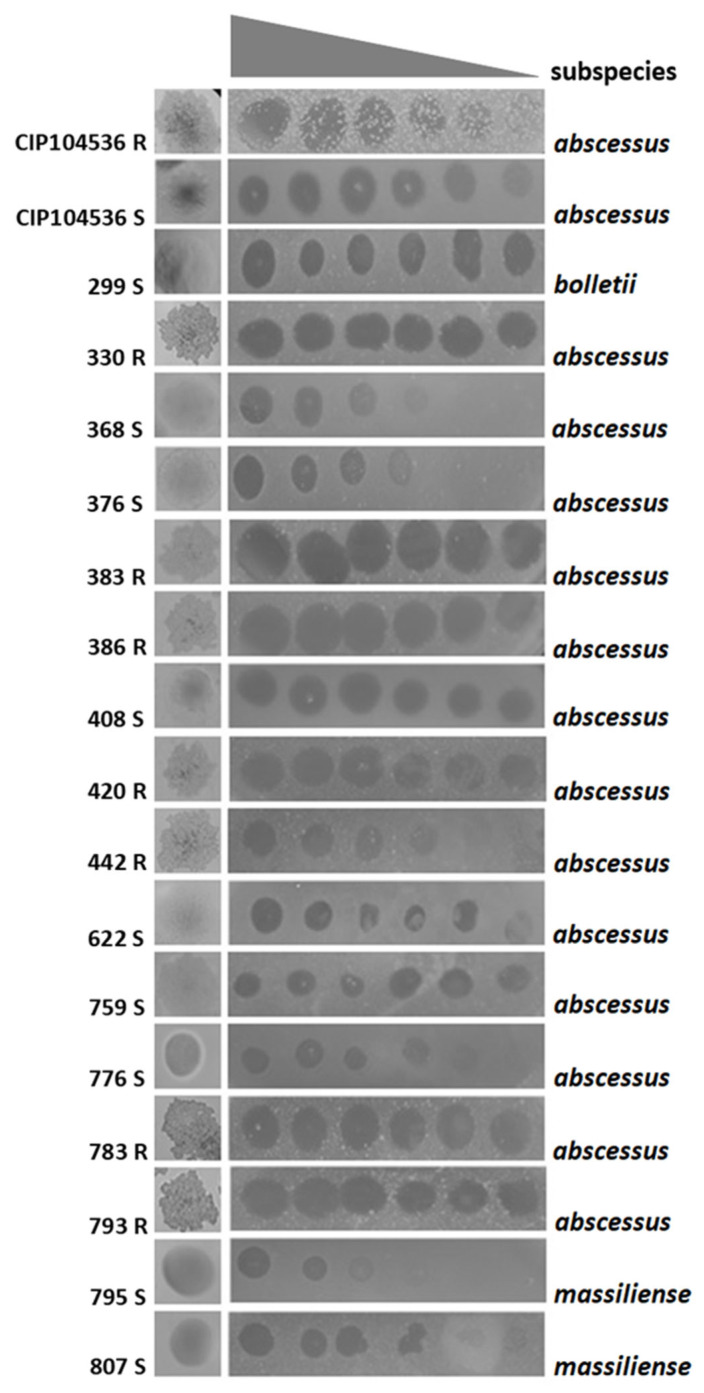
Susceptibility profile of different strains of *M. abscessus* subspecies to P3MA, ranging from 10^10^ to 10^5^ PFU/mL. S: Smooth; R: Rough.

Host range was tested in solid medium using a collection of 30 clinical isolates of *M. abscessus*, containing 21 strains of *M. abscessus* subsp. *abscessus* (30%), 8 strains of *M. abscessus* subsp. *massiliense* (26%), and 1 strain of *M. abscessus* subsp. *bolletii* (4%). P3MA ranging from 10^10^ to 10^5^ PFU/mL infected 16/30 strains (53.3%) of the clinical strains collection: a total of 13/21 *M. abscessus* subsp. *abscessus* strains, 2/8 *M. abscessus* subsp. *masiliense* strains, and 1/1 *M. abscessus* subsp. *bolletii* strain as well as the *M. abscessus* CIP104536T rough (R) and smooth (S) reference strains (Figure 2).

### 2.3. Synergistic Effect of P3MA with Imipenem and Antagonism with Clarithromycin

Checkerboard assays combining P3MA and three antibiotics showed a synergistic effect in the presence of imipenem, with a reduction in the minimum inhibitory concentration (MIC) of the phage and the antibiotic (Table 1). In contrast, combinations with amikacin showed no variations in the MICs of the antibiotic and slightly or no variations in the inhibitory effect of the mycobacteriophage, while an antagonistic effect was observed when P3MA was associated with clarithromycin.

### 2.4. P3MA Inhibits M. abscessus Growth Alone and Combined with Imipenem

**Figure 3:**
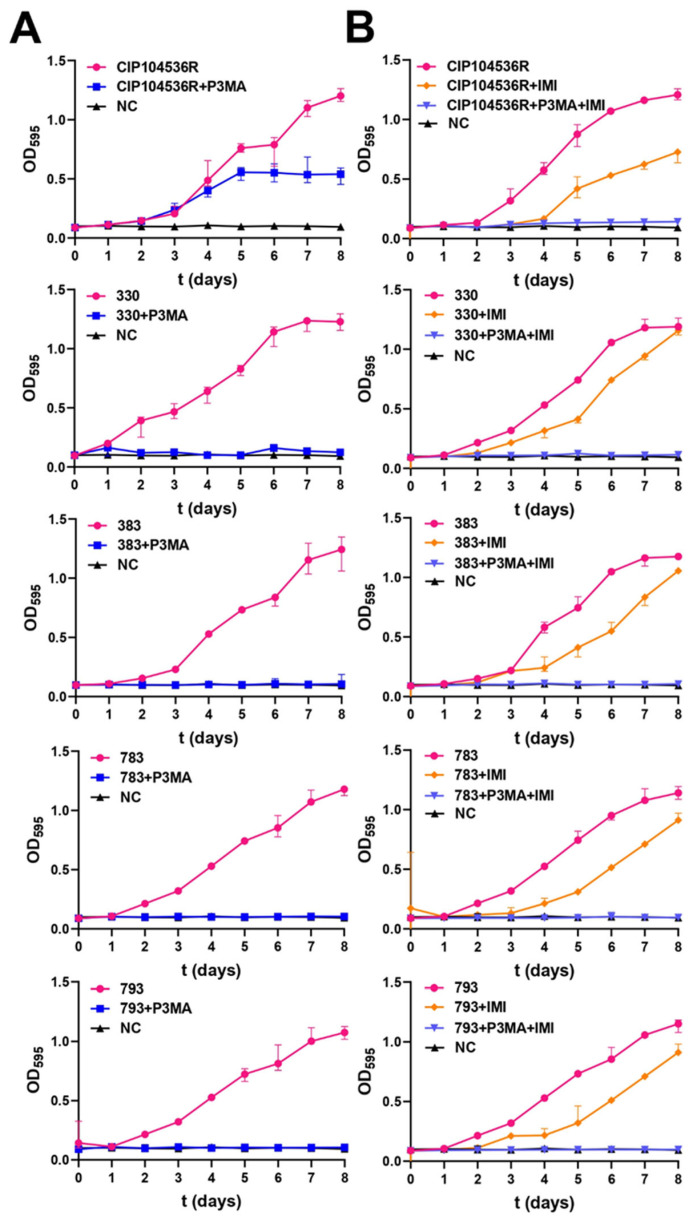
Growth inhibition assays. (**A**): Growth of *M. abscessus* CIP104536T (R) and clinical strains 330, 383, 783, and 793 in the presence of P3MA at MOI 1. (**B**): Growth inhibition effect in the presence of imipenem 14 mg/L and both P3MA (MOI 1) and imipenem 14 mg/L. NC: negative control; OD: optical density; t: time.

Growth curves were performed to analyze the magnitude of the effect of P3MA alone and in combination with imipenem against different clinical *M. abscessus* strains (Figure 3). None of the clinical strains tested (330, 383, 783, and 793) grew at an MOI 1 of P3MA. Only the reference strain CIP104536T (R) showed a delayed growth without reaching the same absorbance as the positive control (growth without phage) (Figure 3A). MOIs 10 and 100 were also preliminarily tested, and they obtained similar results as MOI 1. We chose MOI 1 for the experiments because phages that exhibit strong antibacterial activity at lower MOIs are generally preferred, as they offer advantages overcoming barriers in phage therapy. Interestingly, the synergistic effect of imipenem at 14 mg/L achieved no growth of bacteria for any of the strains tested, including the reference strain (Figure 3B). These results should be interpreted with caution, as imipenem was added only once to the experimental conditions, and this antibiotic is thermolabile at 37 °C [27]. Nevertheless, our findings support that, even with a single dose of imipenem, the phage is capable of completely inhibiting the growth of the tested *M. abscessus* strains.

### 2.5. Mycobacteriophage Effect on M. abscessus Biofilms and a Granuloma-like Model

**Figure 4:**
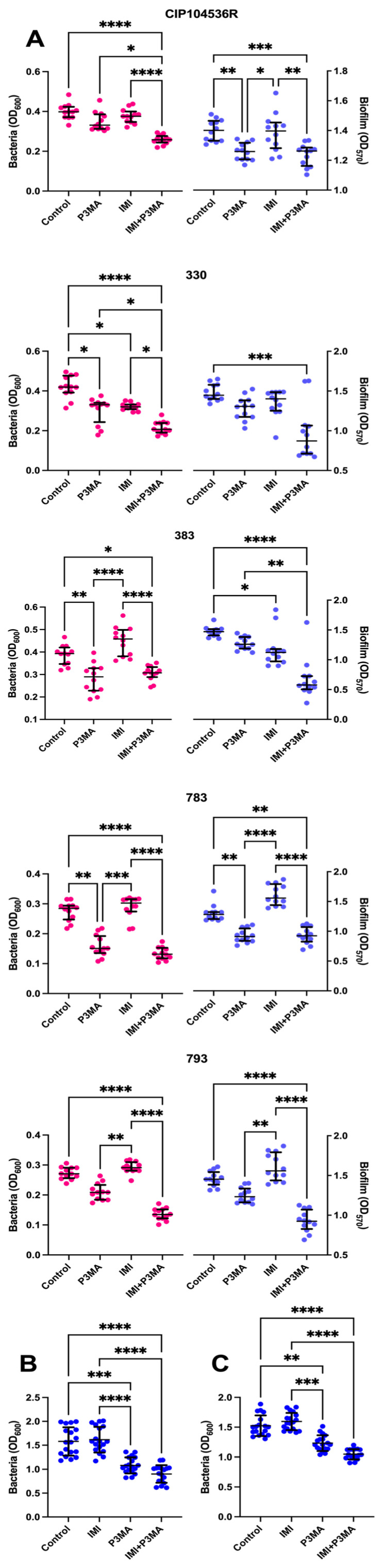
Biofilm assays. (**A**): Planktonic bacteria (pink) derived from the biofilm (blue) of *M. abscessus* CIP104536T (R) and clinical strains 330, 383, 783, and 793 treated with P3MA (MOI 1) with or without imipenem 14 mg/L. The bars represent the median and interquartile range. Treatment of *M. abscessus* biofilm of 783 (**B**) and 793 (**C**) strains grown in granuloma-like medium with bacteriophage P3MA, imipenem 14 mg/mL, and the combination of bacteriophage P3MA and imipenem 14 mg/mL. The bars represent the mean and standard deviation. *: *p*-value < 0.05, **: *p*-value < 0.01, ***: *p*-value < 0.001, ****: *p*-value < 0.0001.

To analyze the effect of P3MA in biofilms, two absorbance measurements were performed. The absorbance at 600 nm represents the inhibition of biofilm growth and the inhibition of planktonic bacteria formation; on the other hand, the absorbance at 570 nm represents the inhibition of biofilm growth only. Treatment of 10^11^ PFU/mL alone against CIP104535T (R) obtained an inhibition of 10.1% of biofilm growth. The same quantity of phage alone against the clinical isolates 330, 383, 783, and 793 showed an inhibition of biofilm growth of 9.7%, 14.4%, 28.8%, and 15.1%, respectively (Figure 4A).

Remarkably, the combination of bacteriophage P3MA at 10^11^ PFU/mL with imipenem at 14 mg/L reached higher levels of inhibition against biofilm growth of clinical isolates than those obtained with the phage alone. Strains 330, 383, 783, and 793 reduced their biofilm growth by 37.8%, 48.5%, 40.7%, and 41.7%, respectively; meanwhile, the CIP104535T strain showed an inhibition of only 9.6% (Figure 4B).

The effect of P3MA against the clinical strains 783 and 793 was tested in a granuloma-like medium (Figure 4C). P3MA (10^11^ PFU/mL) was able to reach a reduction in the *M. abscessus* growth of 31.6 ± 10.6% against the 783 strain and of 19.6 ± 8.7% against the 793 strain. Meanwhile, the combination of P3MA phage at 10^11^ PFU/mL with imipenem at 14 mg/L against the strains 783 and 793 reached 42.9 ± 11.7% and 31.3 ± 5.5% of growth inhibition, respectively (Figure 4C).

## 3. Discussion

*M. abscessus* exhibits high antimicrobial resistance and capacity to form biofilms, making its treatment challenging and causing persistent infections [28]. In this study, a mycobacteriophage named P3MA was characterized in vitro. Typically, the well-known method used to isolate phages infecting mycobacteria uses *M. smegmatis* as an intermediate to isolate new phages against the target species [17]. In this case, the clinical isolate 330 of *M. abscessus* was directly used to find the bacteriophage without the need of using *M. smegmatis* as the target bacterium, and a control plate containing the 330 strain lacking the river water exhibited no calves. However, we cannot ensure the origin of the phage since we have not sequenced the host strain (330), and P3MA is extremely similar to prophage T50-1; on the other hand, P3MA harbors a SNP in an intergenic region that may have produced a lytic derivative. Transmission electron microscopy revealed that P3MA is a siphovirus without an external envelope. As P3MA is like MabB phages, it would be mandatory to delete the toxin and the repressor genes to ensure a lytic cycle and no toxicity prior to use in clinical settings.

P3MA turned out to be stable after one hour of exposure to pH 4.5, 7, and 8, being inactivated at pH 1, similar to other phages infecting other genera [29]. These results revealed that P3MA is not suitable in its natural form for oral administration because of instability at the acidic stomach pH, although it is stable at the slightly alkaline intestinal pH, meaning that gastrointestinal capsules should be considered for the oral route. In addition, P3MA was stable at 4 °C, 21 °C, and −80 °C for one week, being inactivated at 60 °C and losing progressive activity when exposed to −20 °C and 37 °C. This allows the phage to be preserved for short periods; however, recent studies revealed that lyophilization and encapsulation are the best methods to preserve phages [30,31].

P3MA was able to infect 53% of the clinical strains of the collection tested (16 in total), with various efficacies, including smooth (nine strains) and rough strains (seven strains). An approach based on Appelmans’ protocol has recently succeeded in directed evolution against *M. abscessus* clinical strains [32]. Currently, there is no standardization to consider a percentage of host infection as a narrow or broad range [33,34], although usually, authors with phages exhibiting a host range higher than 50% mention they are wide. For clinical application, the highest host range would be desirable in the case of standard cocktails as commercial products; however, for tailored phages in personalized therapies, it is not so important, since phage collections will be tested, or adaptation could be performed.

Antibiotics are usually administered to patients receiving phage therapy [19]. While amikacin and clarithromycin are widely used in *M. abscessus* treatments, our results show an antagonistic effect of P3MA combined with clarithromycin and indifference with amikacin. Interestingly, imipenem exhibited a synergistic effect. Clarithromycin (macrolide) and amikacin (aminoglycoside) are antimicrobials that inhibit the protein synthesis of bacteria. Several authors have mentioned that the protein synthesis inhibition makes it difficult for the virus to replicate, explaining the lack of synergy [35,36]. Although these findings should be tested for more mycobacteriophages, our data suggest that phages and clarithromycin should not be administered together to patients against *M. abscessus* before checking the in vitro interaction in a checkerboard assay to avoid fatal outcomes. On the other hand, synergistic and additive effects are frequently detected in combination with beta-lactams [37]. It has been proposed that bacteriophages can induce mutations in the bacteria, reducing resistance to beta-lactams, such as imipenem [35]; however, the most accepted explanation for synergy comes from the elongation of the cells due to beta-lactams targeting penicillin binding protein involved in the elongosome (such as PBP1a and PBP2 in *E. coli*), which will lead to a higher surface area, increasing the probability that the phage will bind to its target [38,39]. Interestingly, a recent publication reviewed the interactions between phages and antibiotics [40].

Regarding the inhibition of P3MA against planktonic bacteria, no growth of the clinical strains 330, 383, 783, and 793 was detected in the presence of the phage for eight days at an MOI of 1. However, in the case of the reference CIP104536T (R) strain, a delay in growth was observed from the first days of incubation, although it never reached the growth of the positive control lacking phages. Importantly, the combination of P3MA and imipenem (14 mg/L) showed no growth in any of the studied strains. The instability of P3MA detected after 7 days at 37 °C may have diminished the inhibitory effect of P3MA in growth curves, and despite this, the clinical strains tested were inhibited for 8 days, suggesting that a strong initial inhibition could overcome thermal stability at 37 °C.

There is a lack of in vitro studies about the effect of phages against *M. abscessus* biofilms. Our results indicated that the effect of P3MA on the biofilm was notable. It has been described that, for an efficient attack of bacteriophages against biofilms, the biofilm must be immature and challenged by a notable number of bacteriophages [41]. A treatment with the bacteriophage P3MA (10^11^ PFU/mL) was applied to an incipient biofilm of two days of growth of different strains of *M. abscessus*, and a reduction in biofilm growth was detected with P3MA alone; this effect was substantiated when combining P3MA plus imipenem. Although the results may suggest that the phage alone exhibits limited antibiofilm activity, its combination with imipenem significantly enhances this effect. Considering the poor thermal stability of imipenem at 37 °C, the loss of activity observed could be explained by the degradation of the drug over time. Given that the data were obtained using a single dose of imipenem and phage, we hypothesize that multiple or sequential doses could further decrease the viability of biofilm-associated bacteria in rough morphotype strains of *M. abscessus*. The structure of biofilms promotes the coexistence of phage-resistant and phage-susceptible bacteria. Under most conditions, phage-susceptible bacteria are protected from phage predation when growing near to phage-resistant cells [42]. It is important to consider that the observed increase in bacterial viability in the control group may be due to the modest inhibitory effect of phage P3MA on the biofilm growth of *M. abscessus*, which may be intrinsically phage-tolerant and does not produce planktonic bacteria, the form that is susceptible to phage attack [43].

Finally, the granuloma-like medium showed how P3MA, both alone and combined with imipenem at 14 mg/mL, could inhibit the growth of *M. abscessus* in conditions that mimic a granuloma in the hypothetical case that both the mycobacteriophage and imipenem could penetrate the eukaryotic cells. Several studies describe that phages do not penetrate well into eukaryotic cells, although in turn, some cellular mechanisms retaining phages inside eukaryotic cells have been described [44,45,46]. Curiously, in a recent study, the ability of mycobacteriophages to enter mammalian cells and to kill intracellular *M. abscessus* has been described [47]. Our results show that P3MA can inhibit *M. abscessus* both alone and combined with imipenem, achieving significant inhibition. These results also illustrate that P3MA maintains a good bactericidal activity in environmental conditions (granuloma-like medium) very different from those used in the previous procedures, suggesting a promising activity in vivo, although experiments in animals are desirable to study successful outcomes of phage therapy.

## 4. Materials and Methods

### 4.1. Bacterial Strains

Thirty clinical *M. abscessus* strains from patients at the Hospital Universitario Fundación Jiménez Díaz isolated from 2007 to 2020 were studied. These strains were kept frozen at −80 °C in skimmed milk stocks (East Rutherford, Bergen County, NJ, USA) and thawed on TSS blood agar (bioMérieux, Marcy l’Létoile, France) at 37 °C and 5% (*v*/*v*) CO_2_. Identification at the species and subspecies levels was performed by using GenoType ^®^ Mycobacterium CM (Hain Lifescience, Nehren, Germany). The antibiotic susceptibility study was performed by microdilution according to CLSI recommendations using Sensititre™ Myco RAPMYCOI AST (Thermo Fisher Scientific, Waltham, MA, USA).

### 4.2. Bacteriophage Isolation

This procedure was based on the methodology of Van Twest et al. [48]. Phages against *M. abscessus* were isolated from the Manzanares River (Madrid, Spain). Samples were centrifuged at 4500× *g* and filtered with 0.22 µm polyethersulfone (PES) syringe filters (Corning Incorporated, Corning, NY, USA) to remove cell debris. A 100 µL aliquot was incubated in 5 mL of tryptic soy broth (TSB) (Biomérieux, Îlle de France, France) together with the same volume of *M. abscessus* culture of 330 strain (previously incubated for 3 days in TSB) and a final concentration of 10 mM of CaCl_2_ (Thermo Fisher Scientific, Waltham, MA, USA) and 10 mM MgSO_4_ (Thermo Fisher Scientific, Waltham, MA, USA). This culture was incubated for 3 days at 37 °C and 5% (*v*/*v*) CO_2_ with shaking at 80–100 rpm. After centrifugation at 4500× *g* for 20 min, the supernatant was filtered through 0.40 µm and 0.22 µm filters and stored at 4 °C. For phage seeding, the agar-agarose method was used, mixing 3 mL of agarose 0.2% (*w*/*v*) (Panreac Química, Castellar del Vallés, Spain) in TSB containing 10 mM of CaCl_2_ and 10 mM MgSO_4_, 100 µL of liquid culture of 330 *M. abscessus* strain grown for two or three days in TSB, and 100 µL of the previous filtrate. The mixture was poured onto a TSS blood agar plate and incubated for three days. A negative control containing only the 330 strain was also poured to compare calves. Bald spots on the solid medium were cut with a Pasteur pipette; introduced into a 2 mL tube containing 1 mL of sodium magnesium (SM) buffer (SM; 100 mM NaCl (Panreac Química, Barcelona, Spain), 10 mM MgSO_4_ (Thermo Fisher Scientific, Waltham, MA, USA), 10 mM CaCl_2_ (Thermo Fisher Scientific, Waltham, MA, USA), and 50 mM Tris HCl (Sigma-Aldrich, Merck, Darmstadt, Germany), pH 7.5); vortexed 5 min; and centrifuged at 4000× *g* for 5 min. Then, 800 µL of the supernatant was taken and preserved in glass.

### 4.3. Comparative Genomics

The closest nucleotide sequences to P3MA phage (GenBank Accession No. PV089522, already published [24]) from which the complete genome sequence was available were identified using BLASTN. These included phages isolated from two *M. abscessus* subspecies: *abscessus* and *massiliense*. A phage full-genome sequence isolated from *M. abscessus* subspecies *bolletii* was also included for reference. Maximum likelihood (ML) phylogenies were constructed with 1000 ultrafast bootstrap pseudo-replicates as implemented in UFBoot [49] and using the TIM2+F+R3 substitution model chosen according to Bayesian Information Criterion (BIC) by ModelFinder [50] as implemented in IQ-TREE v.2.4.0 [51]. The phylogenetic tree was visualized and edited using FigTree v1.4.5 (http://tree.bio.ed.ac.uk/software/figtree/, accessed on 24 June 2025). Whole-genome average nucleotide identity (ANI) values between P3MA and the phylogenetically nearest phages were estimated using pANIto (https://github.com/sanger-pathogens/panito, accessed on 24 June 2025).

### 4.4. Bacteriophage Enrichment

This method was performed in two similar ways, changing only the liquid and solid culture media. In the first one, one mL of liquid culture of 330 strain in TSB (three days prior incubation) was diluted in 10 mL of liquid TSB medium containing 10 mM CaCl_2_ and 10 mM MgSO_4_ and mixed with 500 µL of phage P3MA. The mixture was incubated at 37 °C at 80–100 rpm for three days. The culture was centrifuged at 4500× *g* and filtered using 0.40 µm and 0.22 µm filters. Serial 1:10 dilutions were made, seeded by the agar-agarose (0.2% (*v*/*v*) of agarose) method on TSS blood agar, and incubated for three days to determine the resulting bacteriophage titer (obtaining around 10^10^ PFU/mL). Large-scale amplification was applied according to the state of the art. In the second method, TSB liquid media were changed to Middlebrook liquid 7H9 with 10% (*v*/*v*) oleic acid, albumin, dextrose, and catalase (OADC), and solid media were changed to Middlebrook 7H10 agar supplemented with OADC. The top agar used was constituted with Middlebrook liquid 7H9 without OADC and 0.7% (*w*/*v*) of BactoAgar. This procedure was based on previous studies [52] and was more efficient (obtaining around 10^12^ PFU/mL).

### 4.5. Bacteriophage Stability Testing

The stability of the isolated bacteriophages was measured in SM buffer at different pH levels (1, 4.5, 7.4, and 8). For this purpose, a 10 µL aliquot of phage was added to 1 mL of SM solution adjusted with HCl and 1M NaOH (Sigma-Aldrich, Castle Hill, NSW, Australia) to reach the desired pH. The samples were incubated for one hour at room temperature. For the thermal stability study, 10 µL of the phage was added to 1 mL of TSB, and the samples were exposed to −80 °C, −21 °C, 4 °C, 21 °C, 37 °C, and 55 °C for 1 h, 24 h, and one week [29].

### 4.6. Bacteriophage Host Range Analysis

The ability of the novel bacteriophages to lyse clinical strains of *M. abscessus* obtained at the Hospital Fundación Jiménez Díaz was checked with a spot test of serial dilutions. This procedure was based on the methodology of Dedrick et al., 2019 [53]. Briefly, an aliquot of each culture was taken, and its optical density was measured at 600 nm. The pellet corresponding to an OD_600_ of 3 was resuspended with 1 mL of Middlebrook 7H9 broth, and 100 µL of this suspension was added to 3 mL of Top agar 7H9 containing 10 mM CaCl_2_ and 10 mM MgSO_4_. The content of the tube was poured into plates of Middlebrook 7H10 agar. Two microliters of diluted P3MA 10^10^–10^5^ PFU/mL was deposited onto agar plates, dried for 20 min, and incubated for three days at 37 °C.

### 4.7. Electron Microscopy

Carbon/colloid-coated copper grids (Gilder Grids, Grantham, UK) with the purified and high titer phage (10^9^ PFU/mL) stocks were negatively stained with 2% (*w*/*v*) uranyl acetate. Images of the sample were taken in a 100 kV JEOL JEM 1011 transmission electron microscope (JEOL). Measurements of phage dimensions were performed with Fiji [54].

### 4.8. Checkerboard

To determine the combined effect of the bacteriophage and an antimicrobial, the checkerboard method was used. A TC treated 96-well plate (Sigma-Aldrich, Merck, Darmstadt, Germany) and cation-adjusted Mueller–Hinton broth (CAMHB) supplemented with 10 mM of CaCl_2_ and 10 mM MgSO_4_ were used, and serial dilutions of both bacteriophage (1:10) and antimicrobials (1:2) were prepared to obtain different combinations between them [55,56]. Phage P3MA concentration ranged from 10^7^ to 10^11^ PFU/mL, imipenem ranged from 0.25 to 64 µg/mL, amikacin ranged from 0.25 to 64 µg/mL, and clarithromycin ranged from 0.5 to 128 µg/mL. The fractional inhibitory concentration (FIC) indexes of imipenem, amikacin, and clarithromycin in combination with P3MA were determined; values ≤ 0.5 were considered synergistic, those from 0.5 to 4 were considered indifferent, and those ≥4 were considered antagonistic. The strains tested in this procedure were *M. abscessus* subsp. *abscessus* CIP104536T (R) and four rough *M. abscessus* subsp. *abscessus* clinical strains, 330, 383, 783, and 793, as they are more typical in clinical settings. The experiment was performed twice for each strain.

### 4.9. Bacteriophage Inhibition Assays

In a treated 96-well plate, a total volume of 200 µL per well contained *M. abscessus* in MDB 7H9 medium supplemented with 1 mM CaCl_2_ and 1 mM MgSO_4_ and the different MOIs of phages. A bacterial growth control without phage was included. In the peripheral wells, 200 µL of sterile water was added to avoid evaporation losses. Absorbance was measured at 595 nm every 24 h for 8 days until the bacterial growth control reached the stationary growth phase. This procedure was based on the methodology used by Wetzel et al., with some modifications [57]. This method was also performed with the combination of bacteriophage and imipenem against the bacteria. The concentration of imipenem used in combination with the phage was 14 mg/L, which corresponds to the concentration of the drug in the lung achieved through an extended perfusion with the lowest dosage of imipenem commonly used in clinical practice [58].

The strains analyzed in this procedure were *M. abscessus* subsp. *abscessus* CIP104536T (R) and the four *M. abscessus* subsp. *abscessus* clinical strains 330, 383, 783, and 793.

### 4.10. Mycobacteriophage Effect on M. abscessus Biofilms

Biofilms were developed using untreated 96-well plates (Thermo Fisher Scientific, Waltham, MA, USA) based on Esteban et al. [59,60]. A bacterial suspension (100 µL OD_600_ 0.1) was inoculated in each well and incubated at 37 °C for 90 min. The suspension was then removed, wells were washed once with sterile phosphate saline buffer (PBS), and 100 µL of CAMHB was added to each well. Plates were statically incubated at 37 °C for 48 h, and supernatants from the wells were discarded. Each well was rinsed once with 100 µL of sterile water, and 100 µL of CAMHB supplemented with 10 mM of CaCl_2_, 10 mM of MgSO_4_, and 10^11^ PFU/mL of P3MA phage were added. Plates were statically incubated at 37 °C for 24 h. OD_600_ was measured after incubation (planktonic bacteria from the biofilm). The supernatant was removed, and each well was rinsed once with 100 µL of sterile water. Fifty microliters of 500 µg/mL of thiazolyl blue tetrazolium bromide (MTT) (Sigma-Aldrich, Merck, Darmstadt, Germany) was added per well. Plates were placed in an orbital shaker (100–150 rpm) and incubated at 37 °C overnight. Then, the reduced MTT was solubilized by adding 100 µL of dimethyl sulfoxide (DMSO) in an orbital shaker (100–150 rpm) and incubated at 37 °C for 1 h. The absorbance of MTT was measured at 570 nm (biofilm bacterial viability). The strains analyzed were *M. abscessus* subsp. *abscessus* CIP104536T (R) and the four *M. abscessus* subsp. *abscessus* clinical strains 330, 383, 783, and 793. This method was carried out with the bacteriophage alone against the bacterial biofilm and with the combination of bacteriophage and imipenem at 14 mg/L against the bacterial biofilm. In both cases, two replicates were carried out. These experiments were performed using six technical replicates and two biological replicates (n = 12).

### 4.11. Granuloma Model

Mycobacterial cells develop drug-tolerant microbial communities that attach to surfaces when cultured in RPMI 1640 medium supplemented with leukocyte lysate over 7 days [61]. These findings strongly suggest that the drug resistance observed in mycobacteria can be attributed entirely to their ability to form biofilms. It is also crucial to highlight that, within caseous necrotic granulomas, extracellular mycobacteria are in contact with leukocyte lysate [62]. The use of this biofilm model may mimic in vitro some of the interactions phages and imipenem can face in vivo. A volume of 100 µL of bacterial suspension with an OD_600_ of 0.1 was deposited on an untreated 96-well plate. The bacterial suspension was prepared in RPMI medium with glutamine supplemented with 2% (*w*/*v*) activated bovine serum and a lysate of 7.6 × 10^6^ mouse monocyte cells [36]. The culture was incubated 24 h in microaerophilia to be treated under four conditions: (i) 200 µL of MHB supplemented with 10 mM CaCl_2_ and MgSO_4_, (ii) 200 µL of phage (10^11^ PFU/mL) in MHB with 10 mM CaCl_2_ and MgSO_4,_ (iii) 200 µL of CAMHB containing 10 mM CaCl_2_ and MgSO_4_ with imipenem at 14 mg/L, and (iv) 200 µL of phage (10^11^ PFU/mL) and imipenem (14 mg/L) in CAMHB containing 10 mM CaCl_2_ and MgSO_4_. CAMHB was chosen with the intention of respecting the imipenem activity. Plates were incubated for 24 h in microaerophilic conditions, and the supernatant was aspirated. Subsequently, 50 µL of MTT (500 µg/mL) was added, and plates were incubated for 24 h [35]. Finally, 100 µL of DMSO was added, and OD_570_ was measured. The strains studied in this procedure were *M. abscessus* subsp. *abscessus* 783 and 793. The procedure was performed using six technical replicates and three biological replicates (n = 18).

### 4.12. Statistical Analysis

All statistical analysis was performed using GraphPad Prism v.8 (GraphPad Prism, version 8.0.1; Windows Version by Software MacKiev © 2020–2018 GraphPad Software, LLC.; San Diego, CA, USA) or R with R Commander (2017).

The distribution of the data was evaluated using the Shapiro–Wilk or Kolmogorov–Smirnov statistics. Descriptive statistics are quoted as median and interquartile range (non-normal distribution) for each variable that was calculated. The Mann–Whitney nonparametric test, considering the equality of variances, was used to compare two groups, and the nonparametric Kruskal–Wallis test was used to compare more than two groups, to determine the effect of temperature on phage viability, and to determine the inhibition of bacterial biofilm by bacteriophages. Statistical significance was set at *p*-values ≤ 0.05. All results are represented as median and interquartile ranges.

## 5. Conclusions

Our results against clinical rough strains point to P3MA, after genetic engineering, as a promising candidate for phage therapy in combination with imipenem for the treatment of lung infections, avoiding the combination with clarithromycin.

## Figures and Tables

**Table 1:** FICI and MIC for amikacin, imipenem, and clarithromycin in the absence or presence of P3MA.

Antibiotic	Strain	ATB (µg/mL) (Phenotype) *	ATB-P3MA (µg/mL)	P3MA (PFU/mL)	P3MA-ATB (PFU/mL)	FICI	FICI Interpretation
Amikacin	CIP 104536 R	32 (I)	32	10^11^	10^11^	2	Indifference
	330	16 (S)	16	10^11^	10^11^	2	Indifference
	383	32 (I)	32	10^11^	10^11^	2	Indifference
	783	16 (S)	32	10^11^	10^11^	2	Indifference
	793	16 (S)	32	10^11^	10^11^	2	Indifference
Imipenem	CIP 104536 R	32 (I)	2	10^11^	10^10^	0.1625	Synergy
	330	64 (R)	2	10^11^	10^10^	0.13125	Synergy
	383	64 (R)	4	10^11^	10^10^	0.1625	Synergy
	783	64 (R)	8	10^11^	10^10^	0.225	Synergy
	793	64 (R)	8	10^11^	10^10^	0.225	Synergy
Clarithromycin	CIP 104536 R	2 (S)	8	10^11^	10^11^	5	Antagonism
	330	4 (I)	32	10^11^	10^11^	9	Antagonism
	383	2 (S)	16	10^11^	10^11^	9	Antagonism
	783	4 (I)	16	10^11^	10^11^	5	Antagonism
	793	4 (S)	64	10^11^	10^11^	17	Antagonism

* Phenotype was determined using the CLSI Standards [26]. ATB: antibiotic; S: susceptible; I: intermediate; R: resistant; FICI: fractional inhibitory concentration index.

## Data Availability

The original contributions presented in this study are included in the article. Further inquiries can be directed to the corresponding author.
